# Book Review: Does Altruism Exist?: Culture, Genes, and the Welfare of Others

**DOI:** 10.3389/fpsyg.2016.01614

**Published:** 2016-10-18

**Authors:** Boróka Pápay

**Affiliations:** “Lendület” Research Center for Educational and Network Studies, Centre for Social Sciences, Hungarian Academy of SciencesBudapest, Hungary

**Keywords:** altruism, group, evolution, culture, genes

Does Altruism Exists? This is an ongoing debate among scientists and philosophers. One of the strongest argument is that every altruistic deed has a selfish motive. “Scratch an altruist and watch a hypocrite bleed,” wrote Michael Ghiselin. David Sloan Wilson, Professor of Biology and Anthropology at Binghamton University seeks an answer this cardinal question. Wilson does not want to introduce new arguments, but rather to present a new approach to the topic. He applies evolutionary perspective to analyze social phenomena and approach altruism. In this framework, altruism is a trait or an evolutionary function of functionally organized groups. In the first five chapters Wilson establishes the theoretical framework used to analyze complex systems such as the economy, religion, or everyday life in the second half of the book to demonstrate how altruism works, what is altruism and why it is so important for humanity.

In the first chapter called “Group That Works” Wilson makes a distinction between altruism at the level of action and at the level of thoughts. An altruistic act may be motivated by a selfish thought and the selfishness of the action would not matter: only its function and its consequences are relevant. Altruism is necessary for a group to function and to be functionally organized. Similarly to insect colonies acting as one super-organism and developing group-level cognition, human societies can be functionally well-organized. Wilson describes well-functioning group using Elinor Ostrom's common pool resources (CPR) theory. There are eight core design principles that sustain CPR groups: strong identity and understanding of the purpose; proportional equivalence between benefits and costs by rewarding members; collective-choice opportunities; monitoring to exclude free riders; gradually implemented sanctions; built-in conflict resolution techniques; minimal recognition of rights to organize for the group itself and for groups belonging to a larger social system; and appropriate coordination between relevant groups. As described in the sixth chapter, most of the world religions create well-organized groups by promoting that every human act is either a win-win, or a lose-lose act. Chapter seven argues that Adam Smith does not mean that individual selfishness creates common good and that unrestrained self interest in economy is harmful.

In the second chapter Wilson claims that an evolutionary story is required to explain functional organization and provides several principles to follow. Firstly, natural selection is based on relative fitness. Secondly, behaving for the good of the group typically does not maximize relative fitness within the group while natural selection operating within the group undermines group-level functional organization. Thirdly, group-level functional organization evolves by natural selection between groups and not within groups. If there is group-level functional organization, altruism exists in terms of action and relative fitness. The third chapter reveals that many theories like evolutionary game theory, selfish gene theory do not invoke group selection. The controversy over the existence of group selection can be solved using equivalence theory stating that ideas, paradigms, and theories deserve to coexist rather than replace each other.

The idea of humans as a society resemble a body or an insect colony comes from Darwin. Humans have distinctive properties. Only basic urges can be explained by biological evolution and the rest is shaped by culture. The fourth chapter is about evolutionary transition of humans. Its foundation is multilevel selection theory that claims that the balance between levels of selection may evolve as well. The chapter explains why humans are the latest transition of evolution and how they evolved from non-humans to humans using social mechanisms. Every major evolutionary transition has three indicators: They happen rarely; they have enormous consequences, for example, when bacteria become super-organism (the level of selection shifts to between groups), it can dominate other, lower-level competitors; when the level of selection shifts to between-group level, the lower levels of functional organizations will disappear partially. Evolutionary transition of humans happened by between-group selection with other primates. Humans successfully, but not completely, suppressed within-group destructive forces by cultivating social control within group competition that benefited the group itself.

The fifth chapter is about the distinction of proximate and ultimate causation in evolutionary theory that can help to reorganize thoughts and feelings that are relevant for the topic of altruism, group-level functional organization at the level of action. Ultimate causation in evolutionary theory is the environmental force that demands adaptation of the organisms that act upon heritable variation causing some traits to evolve. Lactose tolerance, for instance, was triggered by different genes in multiple human groups. Proximate and ultimate causation are complementary to each other and should be studied together. Many proximate mechanisms can result in the same ultimate mechanism. Altruism can be a result of cultural evolution formed by different proximate mechanisms in different cultures, but exists in all of them. As later described in chapter six, every world religion create functionally well-organized groups by different proximate mechanisms. A psychological mechanism is dependent on the environment, including human-constructed environment what decide the evolutionary fate of them. In chapter eight, Wilson reports on his earlier work Binghamton Neighborhood Project, where they measured students' pro-social scores. There was a correlation between the pro-sociality of the individual and the individual's social environment, making pro-sociality spatially heterogeneous. The research team even applied CPR design principles to student groups. The “Pathological Altruism” chapter describes that highly pro-social individuals can show psychological dysfunctionality when they find themselves out of their niche, they can be exploited, or they can be victims of social predators.

Although the theoretical framework is coherent and thought-provoking, familiarizing evolutionary thinking with social scientist, the application of the theory for everyday life yielded mixed results. The attempt to give an explanation for complex systems such as economy or religion were roughly and insufficiently elaborated. The small scale examples, however, described well-demonstrated mechanisms such as in the case of student groups' behavior. The application of CPR designs principles has the potential to make a difference in organizational studies: the understanding of individual traits such as altruism or pro-sociality, and group level phenomena such as between- or within-group competition.

## Author contributions

BP read the book and wrote the review adding her own perspectives.

## Funding

The author participates in a project that has received funding from the European Research Council (ERC) under the European Union's Horizon 2020 research and innovation programme (grant agreement No ERC/648693).

### Conflict of interest statement

The author declares that the research was conducted in the absence of any commercial or financial relationships that could be construed as a potential conflict of interest.

